# Effect of Ultra-High Temperature Degradation on the Physical Properties and Chemical Structure of an AMPS-Based Copolymer Oil-Well Cement Additive PADIM in Aqueous Solution

**DOI:** 10.3390/polym17050591

**Published:** 2025-02-23

**Authors:** Yongjin Yu, Hang Zhang, Pu Xu, Xinyang Zhang, Haige Wang, Miaomiao Hu, Jintang Guo

**Affiliations:** 1 CNPC Engineering Technology R&D Company Limited, Beijing 102206, China; yuyongjindri@cnpc.com.cn (Y.Y.); wanghaigedri@cnpc.com.cn (H.W.); 2 National Engineering Research Center of Oil & Gas Drilling and Completion Technology, Beijing 102206, China; 3 School of Chemical Engineering and Technology, Tianjin University, Tianjin 300350, China; 18703458175@163.com (H.Z.);; 4 Zhejiang Institute of Tianjin University, Shaoxing 312300, China; 5 Haihe Laboratory of Sustainable Chemical Transformations, Tianjin 300192, China

**Keywords:** oil-well cement, copolymer fluid loss agent, ultra-high temperature, thermal degradation, functional group transformation

## Abstract

2-acrylamido-2-methylpropane sulfonic acid (AMPS) based copolymer fluid loss agent is a kind of widely utilized additive in oil-well cement. However, when applied in ultra-high temperature (UHT) formation environment, its fluid loss control efficiency is significantly declined due to the thermal degradation behavior, and corresponding mechanism study is still lacking. Regarding the above issue, this work synthesized one representative copolymer fluid loss agent PADIM and investigated its thermal degradation mechanism in UHT aqueous environment, which was polymerized by AMPS, N, N-dimethylacrylamide (DMAA), itaconic acid (IA) and methacryloxyethyltrimethyl ammonium chloride (MTC). The aim of this paper was to provide a theoretical guidance for the futural structural design of the fluid loss agents for oil well cement slurry at UHTs. The copolymer solution was subjected to isothermal aging at 180–240 °C for 1.5 h or 6.0 h (to simulate short-period and long-period aging, respectively), and the aged products were further analyzed. It was found that the thermal decomposition onset temperature of the copolymer solid was 294.6 °C. However, its thermal stability in aqueous solution was significantly lower, with substantial main chain breakage and functional group transformations occurring below 240 °C. As a result, the apparent viscosity and average molecular weight were significantly reduced from 4216 mPa·s and 31,666 Da before aging to 107.4 mPa·s and 8590 Da after aging at 240 °C for 6.0 h. Meanwhile, the side groups (-SO_3_^−^ and -COO^−^) were removed and the unsaturated alkenes were produced due to main chain degradation. In terms of application performance, the fluid loss control ability of the aged product diminished gradually from 22 mL to 196 mL as the aging temperature increased from room temperature to 210 °C. This decline was attributed to a reduction in molecular weight and a decrease in product adsorption capacity caused by the removal of side groups.

## 1. Introduction

Oil-well cement is an important material in the construction process of oil and gas wells, and its performance directly affects the long-term stability and production efficiency of oil and gas wells. In recent years, the exploitation of oil and gas resources has gradually advanced toward ultra-deep formations with depths reaching 8000–10,000 m [1]. The elevated downhole circulating temperatures and pressures pose significant challenges to the development of cement additives for wellbore stabilization [2,3]. In these harsh and complex environments, the fluid loss phenomenon of oil-well cement slurry would intensify, leading to the decrease of the cementing quality and even affecting the life of the oil well [4,5]. During the dehydration process of cement slurry, the water escapes from the slurry through various pathways under capillary action, pressure drive, and chemical adsorption, etc. Under high temperature conditions, the water loss rate of cement slurry would significantly accelerate, excessive water loss could cause the following hazards: (1) the rheological property of cement slurry decreases obviously, affecting the operability of construction; (2) The hydration reaction of the cement slurry is hindered due to a large amount of water loss, resulting in a decrease in its sealing effect on the wellbore; (3) The decline in cementing quality may lead to engineering problems such as well leakage and blowout. Therefore, research and development of high-temperature resistant fluid loss agents have become an important topic in the field of oil-well cement technology. The fluid loss agent is one of the indispensable oil-well cement additives, which is used for preventing the slurry from infiltrating formation fractures [6,7]. However, the increasing well depth and temperature have imposed more stringent requirements on the thermal stability of these agents [8,9,10].

Currently, the most commonly used fluid loss agents for oil-well cement include particulate materials, starch derivatives, cellulose derivatives, synthetic copolymers and so on [11,12,13,14,15]. The limited temperature resistance of the first three materials restricts their extensive applications. In contrast, artificially synthesized polymers, as a kind of multifunctional material with wide application characteristics [16,17], are often added to oil well cement as performance control additives because their properties can be independently regulated [18]. During which, synthetic AMPS-based copolymers are predominantly used as high-temperature fluid loss agents. Common monomers for these copolymers include amide-based monomers (e.g., AM, DMAA) and carboxylic acid-based monomers (e.g., MAH, IA) [8,19,20]. The mechanism of action of copolymer fluid loss agents primarily includes three aspects [21,22,23]: (1) Adsorption effect: the hydrophilic groups in the additive adsorb onto the surface of cement particles, forming a layer that reduces water diffusion and decreases fluid loss. (2) Viscosity enhancement: the additive increases the viscosity of the cement slurry, thereby reducing the infiltration rate of liquid into filter paper or the formation. (3) Film-forming effect: the copolymer forms a membrane that coats solid particles, reducing the porosity of the filter cake and obstructing fluid loss.

Research has shown that while AMPS-based fluid loss agents could exert partial effect in ultra-deep formation environments with temperatures up to 200 °C [2,7], but the low dosages are usually unable to control the fluid loss effectively [24,25]. Therefore, it is often necessary to significantly increase the dosage to meet actual requirements. Excessive amount of introduction of fluid loss copolymers can lead to a substantial decline in the stability and thickening performance of the slurry [26]. Under high-temperature and high-pressure conditions, copolymer fluid loss agents are prone to experience the thermal oxidative degradation, resulting in main chain breakage and side group variations. These degradation phenomena would cause structural aging of the copolymer, and thus compromise its application performance [27,28]. The cracking of the main chain is usually caused by the presence of dissolved oxygen, which stimulates the generation of peroxide radicals in the polymer [29,30]. The peroxide radicals gradually break the molecular chain and produce low molecular weight products, thereby resulting in the loss of polymeric functionality. The side group variations include the hydrolysis or removal of amide groups and carboxyl groups contained in fluid loss agents at high temperature, which would cause the desorption of anionic anchoring groups and thus leading to the deterioration of fluid loss control capacity. Therefore, studying the thermal degradation mechanism of water loss agents under ultra-high temperature conditions is of great theoretical significance for future research on improving their temperature resistance through molecular structure design methods.

To elucidate the high-temperature degradation mechanism of copolymer fluid loss agents in aqueous environments, this study employed a sealed metal aging tank to conduct thermal degradation experiments. The fluid loss agent solution was placed inside the tank and pressurized with nitrogen to prevent boiling at high temperatures. The system was then heated to 180–240 °C for aging experiments. The resulting products undergo various microstructural characterizations to comprehensively analyze the evolution of the physical properties, molecular structure, and application performance of the copolymer fluid loss agent under ultra-high temperatures. These findings aim to provide theoretical references for the subsequent structural design and product development of ultra-high temperature cement additives.

## 2. Experimental

### 2.1. Materials and Methods

#### 2.1.1. Materials

2-acrylamido-2-methylpropane sulfonic acid (AMPS) was purchased from Borun Chemical Co., Ltd. (Dezhou, China). Sodium hydroxide (NaOH), potassium persulfate (KPS) and itaconic acid (IA) were acquire from Dibo Technology Co., Ltd. (Shanghai, China). N, N-Dimethylacrylamide (DMAA) and methacryloxyethyltrimethyl ammonium chloride (MTC) were received from Damao Chemical Reagent Co., Ltd. (Tianjin, China).

#### 2.1.2. Research Methodology

**Figure 1:**
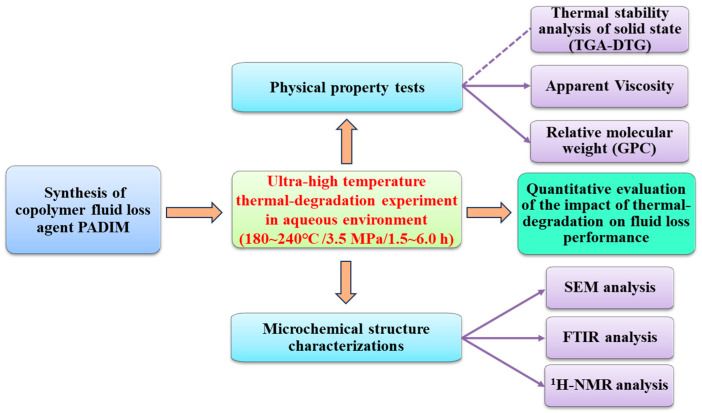
The research methodology roadmap of this work.

In this work, the thermal-degradation mechanism of the representative copolymer fluid loss agent PADIM [P(AMPS/DMAA/IA/MTC)] in UHT aqueous environment was studied based on the analyses of physical properties, chemical structure and performance evolution. The research methodology roadmap was presented in Figure 1.

### 2.2. Tests and Characterizations

#### 2.2.1. Synthesis of the Copolymer PADIM

**Figure 2:**
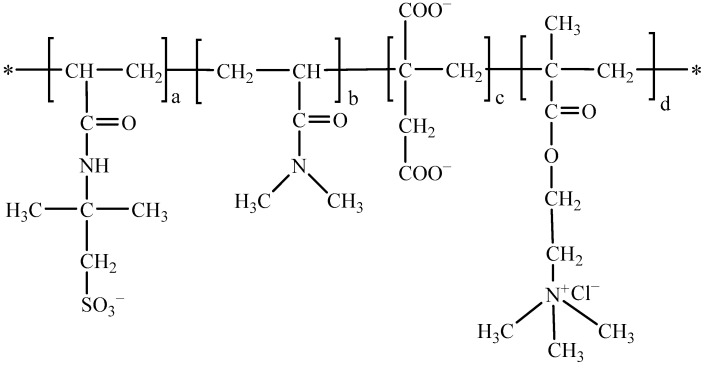
Molecular structure of the copolymer PADIM.

The high-temperature fluid loss agent PADIM was synthesized using an aqueous solution free-radical polymerization method. The procedure was as follows: Firstly, AMPS and IA were dissolved in deionized water, and the pH of the system was adjusted to 6 using 35 wt% NaOH solution. Then, the monomers DMAA and MTC were added and the mixture was stirred evenly. Finally, the solution was transferred into a four-neck flask preheated to 60 °C, and the initiator KPS was added. After stirring for 30 min, the temperature was gradually increased to 80 °C, and the reaction was maintained for 2.5 h. The final prepared PADIM had a solid content of 20 wt%, and its chemical structure was shown in Figure 2.

#### 2.2.2. Ultra-High Temperature Thermal Degradation Experiments

In this work, except for the TGA-DTG analysis that was performed directly on the solid copolymer PADIM, all other characterization/test samples of the thermal degradation products of PADIM were obtained from the following aging experiments in aqueous environment. Firstly, the purified copolymer solution was placed into a sealed metal aging tank and the system was pressurized to 3.5 MPa using N_2_ to avoid liquid boiling at 240 °C. Then, the thermal degradation experiments were conducted at target temperatures for 1.5 h or 6.0 h to respectively simulate the shorter or longer time aging. Finally, the aged products were freeze-dried for subsequent analyses.

#### 2.2.3. Physical Property Tests

(1) Thermal stability

The thermal stability of the solid copolymer PADIM was characterized using a TGA-Q500 thermogravimetric analyzer (TGA). The purified polymer powder (0.002–0.005 g) was added into an alumina crucible. The TG-DTG curves were measured at a temperature range of 35–600 °C with a heating rate of 10 °C/min, and the N_2_ was applied as the protective gas with a flow rate of 30 mL/min.

(2) Apparent viscosity

The apparent viscosity of 20 wt% PADIM solutions and its thermal degradation product solutions at different temperatures were measured at room temperature using a rotational viscometer (Model DV2T, Brookfield company, Middleboro, MA, USA).

(3) Relative molecular weight

The molecular weight of copolymer PADIM and its aged products in aqueous solution were tested using gel permeation chromatograph (GPC) technique (Model TDA305, produced by Malvern Instruments co. Ltd., Malvern, UK). The fluid loss agent samples were dissolved in a 0.1 mol/L sodium nitrate solution as the mobile phase with a flow rate of 0.5 mL/min.

#### 2.2.4. Microchemical Characterizations

First, the chemical structure of the dried polymer powder was characterized using a Fourier-Transform Infrared spectrometer (FTIR, Model Bio-Rad FTS 3000, Hercules, CA, USA) with the wave number range of 400–4000 cm^−1^. Then, the dried polymer sample power was dissolved in deuterium oxide (D_2_O) and characterized using a Nuclear Magnetic Resonance (NMR) spectrometer (Model Avance III 400 MHz, Bruker, Germany).

#### 2.2.5. Static Fluid Loss Test

A single-factor quantitative evaluation method (temperature) was used to investigate the impact of ultra-high temperatures on the performance of the fluid loss agent. Specifically, the samples aged at ultra-high temperatures (180–240 °C) were tested at a consistent dosage (4% by weight of cement) and temperature (90 °C) for fluid loss performance. The data were used to quantitatively evaluate the application performance of the products. The testing procedure was based on the guidelines outlined in Section 9.3 of the API Recommended Practice 10B-2 [31].

## 3. Results and Discussion

### 3.1. Evolution Mechanism of Physical Properties

#### 3.1.1. Thermal Stability Analysis

**Figure 3:**
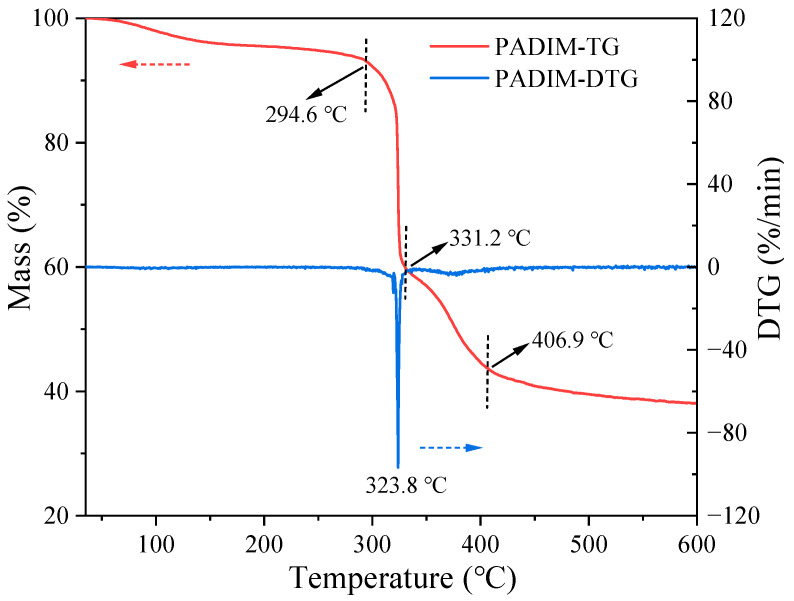
TG-DTG curves of the PADIM copolymer.

The TG-DTG characterization result of the solid copolymer PADIM was shown in Figure 3. It could be observed that in the temperature range of 35–120 °C, the mass loss was approximately 3%, which was attributed to the evaporation of a small amount of free water contained in the sample [32]. In the temperature range of 120–294 °C, the mass loss rate was around 4.1%, which could be due to the removal of side groups such as amide, sulfonic acid, and carboxylic groups from the copolymer [4], while the main chain of the copolymer may have broken into shorter chain fragments that have not yet volatilized. When the temperature reached 294.6 °C, PADIM began to decompose intensely, reaching its maximum thermal decomposition rate at 323.8 °C. Within the range of 294.6–331.2 °C, the mass loss reached 33.4%, which represented that the degraded copolymer fragments of main chain decomposed into small molecular monomers and volatilize [33]. In the range of 331.2–406.9 °C, the sample continued to decompose with a further mass loss of 16.1%, signifying the carbonization process occurred among these temperature range.

The TG-DTG analyses indicated that the solid PADIM copolymer exhibited relatively high structural thermal stability with the onset temperature of main chain thermal decomposition (294.6 °C) significantly exceeding its practical application temperature [28]. According to the TGA test results of AMPS-type fluid loss agent in the existing literatures, the thermal stability of solid copolymer fluid loss agent is generally high, with the thermal decomposition temperature reaching 300 °C or above [34,35]. Given the degradation pathway of solid sample under N_2_ atmosphere differed significantly from its behavior in aqueous environments containing dissolved oxygen, further investigations in aqueous degradation were required to be performed.

#### 3.1.2. Apparent Viscosity Analysis

**Figure 4:**
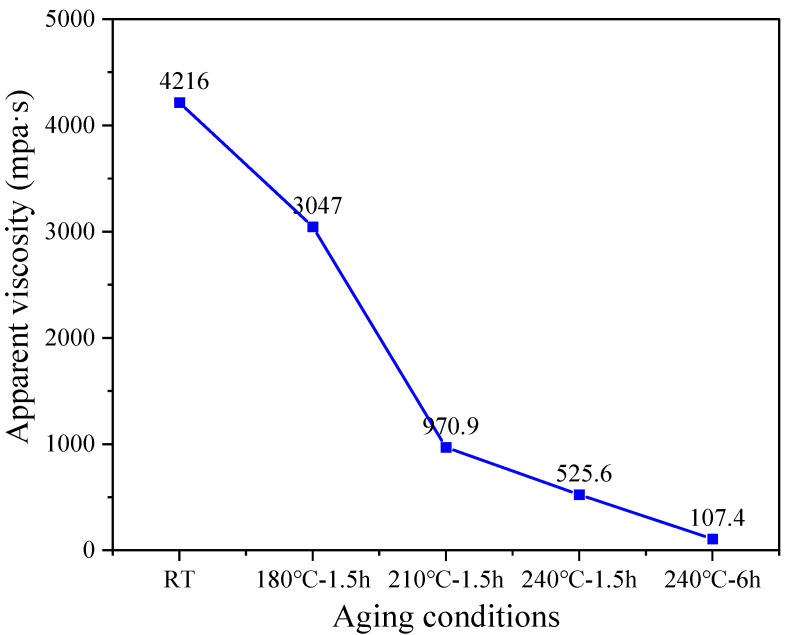
Apparent viscosity of the copolymer PADIM degraded at different temperatures.

The apparent viscosity of the PADIM copolymer degraded at different temperatures was shown in Figure 4. As could be seen, the apparent viscosity of the PADIM solution at room temperature (RT) was 4216 mPa·s. After degradation at 180 °C for 1.5 h, the viscosity decreased to 3047 mPa·s (reduced 27.7% compared to RT). When the aging temperature continued to increase to 210 °C for 1.5 h, the viscosity sharply decreased to 970.9 mPa·s with a 68.1% reduction compared to 180 °C, indicating that the main chain breakage of PADIM in solution become increasingly severe above 180 °C. Further degradation at 240 °C for 1.5 h and 6.0 h led the viscosity decline to 525.6 mPa·s and 107.4 mPa·s, respectively. At this temperature, the molecular thermal motion of polymer chains was enhanced, and the active bonds with low bond energy (such as C-C, C-O, C-S, etc.) [29,36] were broken due to thermal excitation, resulting in a significant decrease in the molecular weight of thermal degradation products. Due to the positive correlation between the apparent viscosity and molecular weight of linear copolymers, thereby the apparent viscosity was obviously lost (97.5% compared to RT).

#### 3.1.3. Relative Molecular Weight (Mn) Analysis

**Figure 5:**
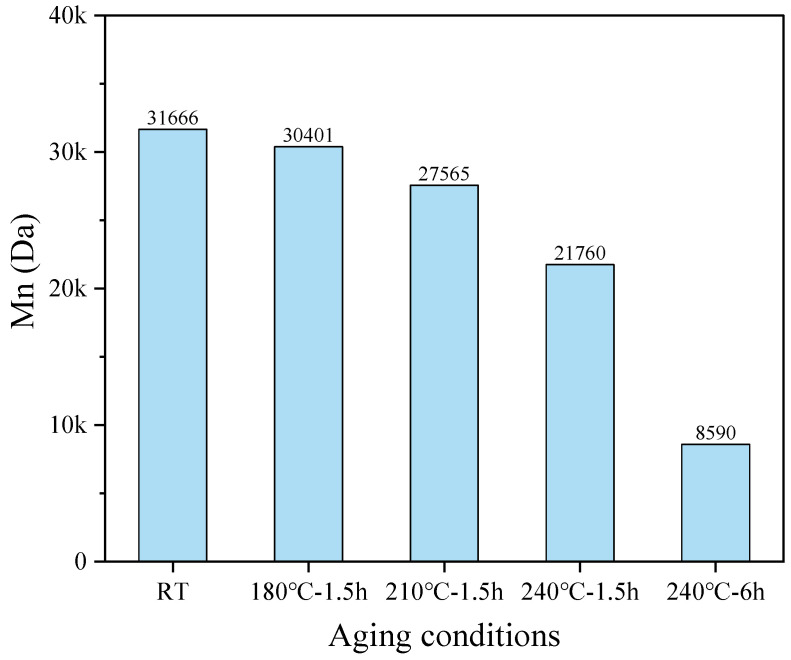
Relative molecular weight M_n_ of the copolymer PADIM degraded at different temperatures.

The relative molecular weight of PADIM after thermal degradation at different temperatures was determined and shown in Figure 5. As could be seen, the number-average molecular weight (M_n_) of PADIM before degradation was 31,666 Da. As the aging temperature increased, the M_n_ values of the degradation products decreased progressively, and more significant reductions were observed when temperature reached above 210 °C. Specifically, the (M_n_ of the sample aged at 240 °C for 1.5 h was 21,760 Da, representing a 21.1% reduction compared to the sample “210 °C–1.5 h” (27,565 Da). Further aging at 240 °C for 6.0 h reduced the molecular weight by 60.5% (from 21,760 Da to 8590 Da) compared to “240 °C–1.5 h” sample. Combined with the analysis in the following text (Section 3.2), it could be concluded that the hydrolysis of amide group in AMPS and the decarboxylation reaction of IA would both lead to the removal of PADIM side groups. In addition, the cracking of the copolymer main chain would release unsaturated alkenes. The two features mentioned above were the main factors leading to the decrease in M_n_ of the thermal degradation products of PADIM.

In summary, PADIM copolymer solutions underwent mild thermal oxidative degradation in the temperature range of 180–210 °C, and this behavior became more severe at 240 °C accompanied by many small molecular weight fragments generated. Therefore, the unaged PADIM sample at RT as well as the degradation products aged at 180 °C and 240 °C were selected for subsequent chemical structure characterizations.

### 3.2. Evolution Mechanism of Microchemical Structure

#### 3.2.1. SEM Micro-Morphology Analysis

**Figure 6:**
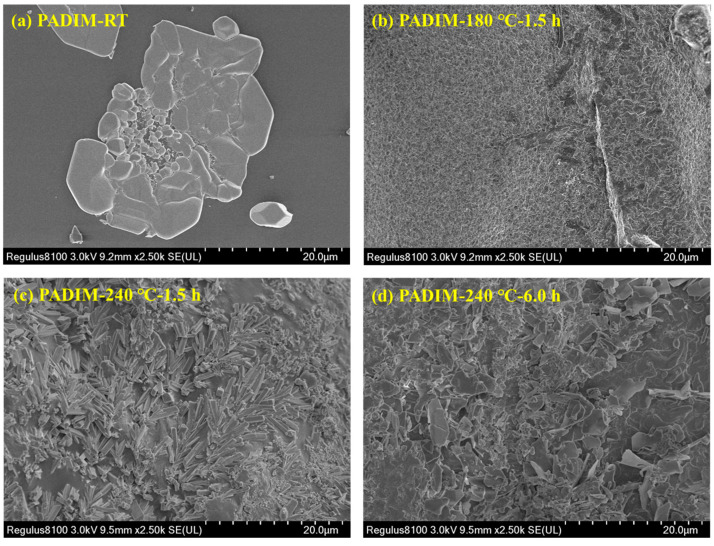
SEM images of copolymer PADIM and its degradation products at different temperatures.

SEM was firstly applied to observe the micromorphology of freeze-dried copolymer PADIM and its degradation products at different temperatures, the results are shown in Figure 6. It could be seen that the copolymer PADIM-RT (before aging) exhibited a flat film-like structure. After aging at 180 °C for 1.5 h, this flat structure was destroyed and many tiny cracks were formed. When the aging temperature further rose to 240 °C and aged for 1.5 h, an interesting phenomenon occurred where the PADIM morphology evolved into a rod-shaped crystal structure. As the aging time was further extended to 6.0 h at 240 °C, the PADIM morphology evolved into a multi-fragment structure. From the above analysis, it could be concluded that after undergoing ultra-high temperature aging in aqueous solution, the thermal degradation behavior of copolymer PADIM led to a significant variation of its chemical structure, thereby changing the micromorphology of the degradation product. In the following sections, microstructural characterization techniques would be introduced to further analyze the chemical structure evolution of thermal degradation products.

#### 3.2.2. FTIR Analysis

**Figure 7:**
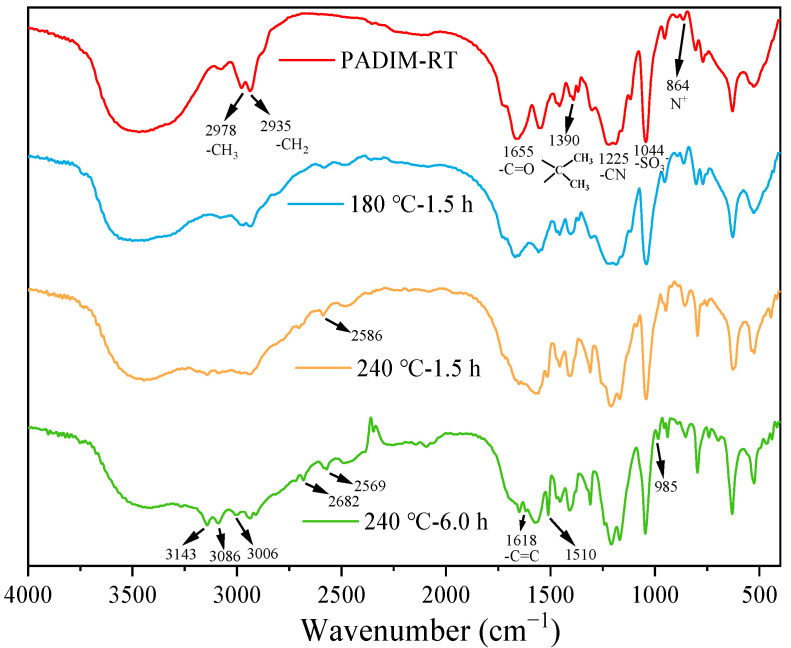
FTIR spectra of copolymer PADIM and the degradation products at different temperatures.

The FTIR spectra of copolymer PADIM and its degradation products aged at 180 °C and 240 °C are shown in Figure 7. As was shown, for the PADIM copolymer and its products aged at 180 °C and 240 °C for 1.5 h, no significant changes in peak shape were observed. However, after aging at 240 °C for 6.0 h, new peaks appeared near 3143 cm^−1^, 3086 cm^−1^, and 3006 cm^−1^ which corresponded to the stretching vibrations of C-H bonds on C=C double bonds [37]. Additionally, a characteristic peak for C=C double bonds appeared at 1618 cm^−1^, indicating severe thermal oxidative degradation of the copolymer [38,39]. Moreover, the peaks newly appeared at 2509 cm^−1^ and 2682 cm^−1^ were attributed to substituted -NH_2_ groups due to the hydrolysis of AMPS [27,40]. Meanwhile, the N^+^ peak at 864 cm^−1^ disappeared, suggesting that the ester group in MTC underwent hydrolysis and thus resulting in the removal of the quaternary ammonium functional side group. Combined with the viscosity analysis, it was obvious that short-term aging at ultra-high temperatures (1.5 h) primarily resulted in main chain breakage without significant structural changes. In contrast, more prolonged aging (6.0 h) at 240 °C would cause substantial structural variations and produce large quantities of unsaturated alkene structures [38,41]. The results obtained from FTIR analyses were consistent with the existing studies on the thermal degradation of AMPS-based copolymers [27,38,41].

##### 3.2.3. ^1^H-NMR Analysis

**Figure 8:**
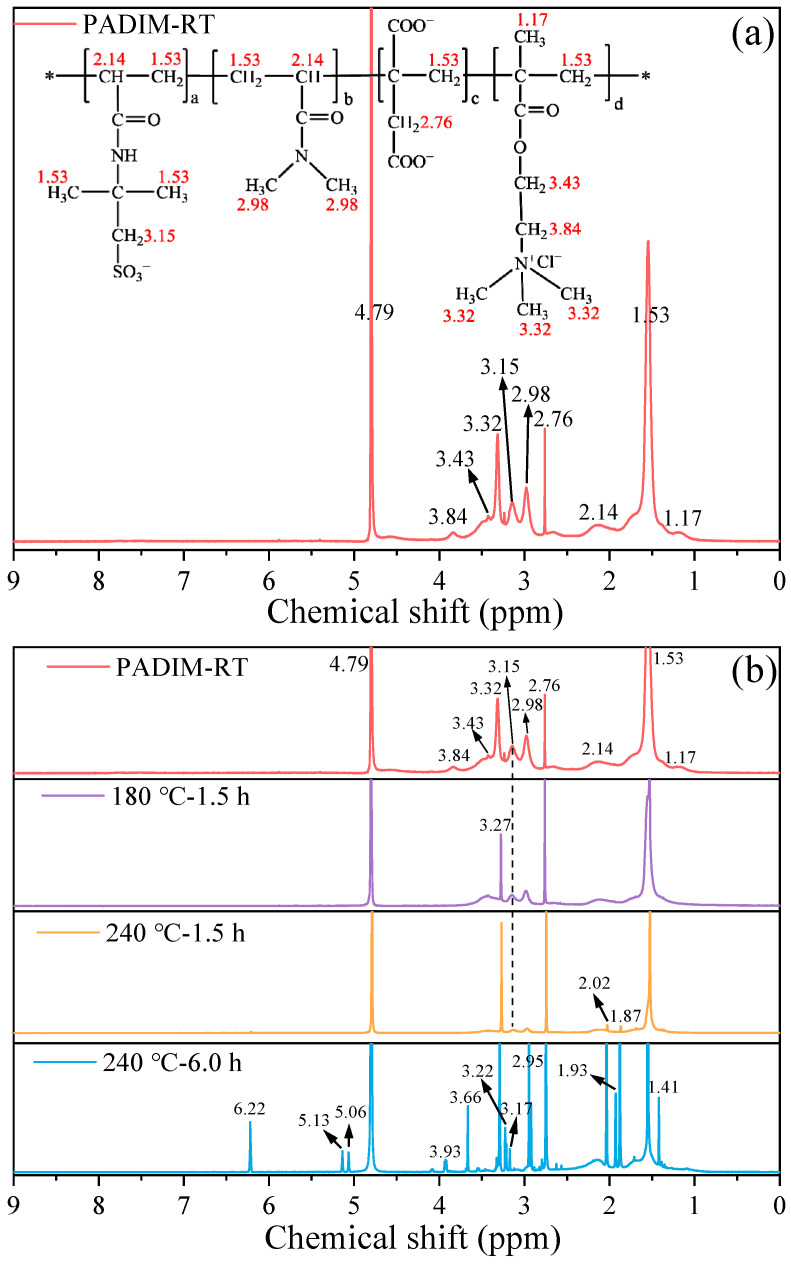
^1^H-NMR spectra of (**a**) copolymer PADIM and (**b**) its degradation products at different temperatures.

This study further utilized the more sensitive ^1^H-NMR spectroscopy to perform a microstructural characterization of PADIM samples before and after thermal degradation, the results were presented in Figure 8. As was shown, the peak at a chemical shift of 1.53 ppm corresponded to the characteristic hydrogens of -CH_2_ in the polymer main chain and -CH_3_ in AMPS [42], and the peak at 2.14 ppm was associated with the characteristic hydrogens of -CH in the polymer main chain [43]. Moreover, 2.76 ppm was assigned to the characteristic hydrogens of -CH_2_ in the IA side chain, while 2.98 ppm represented the characteristic hydrogens of -CH_3_ connected to a nitrogen atom in DMAA [22]. Furthermore, 3.15 ppm was attributed to the -CH_2_-SO_3_ structure in the AMPS side chain. As for the functional monomer of MTC, the peak at 1.17 ppm is attributed to the -CH_3_ connected to the main chain; the peak at 3.32 ppm belonged to the -CH_3_ connected to the N^+^ ion; the peaks at 3.43 ppm and 3.84 ppm are associated with two -CH_2_ groups linked to -COO⁻ in the MTC side chain.

As was shown in the spectra, when PADIM was aged at 180 °C for 1.5 h, only the peaks in the range of 3.32–3.84 ppm shifted or disappeared, which indicated that the ester groups in the MTC side chain underwent hydrolysis and led to the removal of side groups. For the “240 °C–1.5 h” sample, two new characteristic peaks appeared at 1.87 ppm and 2.02 ppm, corresponding to the hydrogens of methylene groups linked to double bonds (-CH_2_-C=C) generated by slight degradation. However, in the “240 °C–6.0 h” sample, numerous new peaks including a new peak at 6.22 ppm appeared, which corresponded to the hydrogens on -CH=CH_2_ double bonds [38,44]. Moreover, it could be observed that two characteristic peaks at 2.98 ppm and 3.15 ppm disappeared and some new sharp peaks representing small molecules generated. This result indicated that both AMPS and DMAA underwent hydrolysis accompanied by the sulfonic groups and methyl groups removed. In summary, when the aging temperature reached 240 °C, the polymer configuration underwent significant transformations. Specifically speaking, When the thermal degradation time was prolonged, a large number of unsaturated C=C double bonds would be generated meanwhile the side groups of AMPS and DMAA would be removed.

### 3.3. Evolution Tendency of Fluid Loss Performance

**Figure 9:**
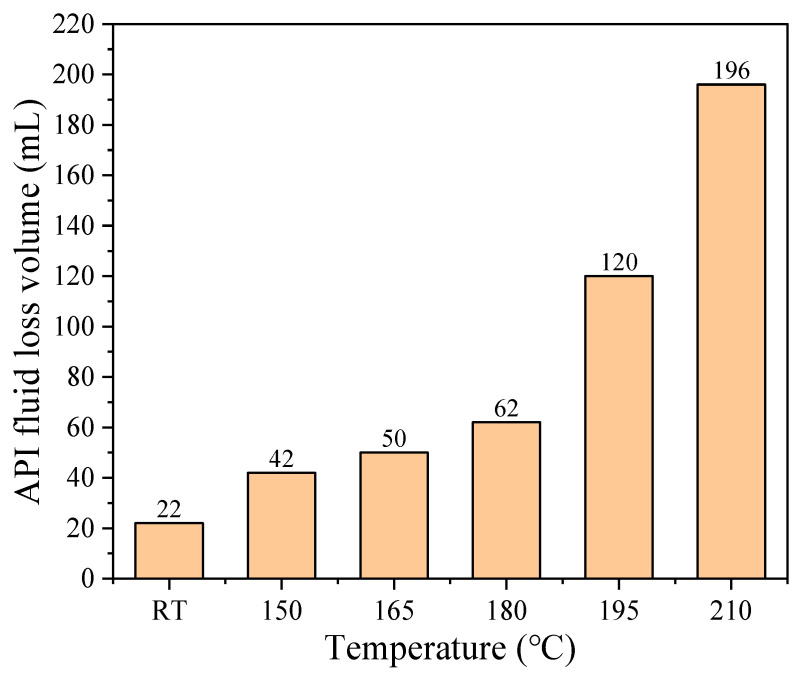
Effect of different aging temperatures on the fluid loss control performance of PADIM.

The static fluid loss performance of the copolymer PADIM and its thermally aged products was tested in cement slurries at 90 °C, the results were shown in Figure 9. Results showed that the fluid loss control efficiency of the copolymer decreased gradually as the aging temperature raised. However, the performance of the aged product still met the practical requirements at aging temperatures below 180 °C. When the aging temperature reached to 195 °C and above, the fluid loss efficiency deteriorated significantly with API fluid loss volumes exceeding 120 mL, making it unsuitable for cementing operations. By comparing with existing literature on copolymer fluid loss agents, it could be found that the variation tendency of determined fluid loss volume was consistent with the results of the temperature-dependent performance of other copolymer fluid loss agents reported in relevant literatures [45,46,47]. This result indicated that copolymer fluid loss agents generally suffer from the effectiveness decrease at ultra-high temperatures. Therefore, it is necessary to conduct research in this paper to explore the evolution of physical properties and chemical structures in ultra-high temperature environments.

Combined with the microstructural analysis discussed above, it could be concluded that the severe thermal oxidative degradation and structural alterations of PADIM above 200 °C were the main reasons for this deterioration. The significant decline in viscosity and molecular weight along with the removal of anonic side groups such as -COO⁻ and -SO_3_^−^ weakened the adsorption ability of the degraded product, thereby impairing its fluid loss control performance. These findings suggested that future designs for ultra-high-temperature fluid loss agents should focus on improving the molecular architecture to overcome the limitations of current AMPS-based copolymers.

## 4. Conclusions

This study investigated the evolution mechanism of physical properties, microchemical structure and application performance of a representative AMPS-based copolymer fluid loss agent PADIM in ultra-high temperature aqueous environment, in order to reveal the reason for the decrease of copolymer’s fluid loss reduction efficiency at ultra-high temperature from the molecular level. The following conclusions could be drawn:

(1) Within ultra-high temperature range of 180–240 °C, the copolymer PAIDM exhibited a progressive decrease in apparent viscosity and molecular weight, as their parameters were significantly reduced from 4216 mPa·s and 31,666 Da before aging to 525.6 mPa·s and 21,760 Da after aging for 1.5 h at 240 °C. This determination results reflect that the copolymer gradually underwent severe thermal oxidative degradation in ultra-high temperature aqueous environment, and this process accelerated as the temperature raised. The decrease of the apparent viscosity and molecular weight of the copolymer was the first factor leading to the decrease of its performance at ultra-high temperature.

(2) The FTIR and ^1^H-NMR characterizations demonstrated that the copolymer PAIDM underwent main chain degradation and the variation of functional groups after exposure to ultra-high temperature aqueous environment. As a result, the side groups such as -SO_3_^2−^ and -CH_3_ were removed after short time aging (1.5 h). Further extension of aging time to 6.0 h exacerbated the degradation extent and broke the PADIM main chain, accompanied by the generation of unsaturated alkenes. Therefore, the main chain structure deviated from its original form was the other significant factor causing its performance deterioration at ultra-high temperature.

(3) The quantitative test of fluid loss performance of PADIM and its aging products indicated that the effectiveness of PADIM deteriorated significantly when the aging temperature reached 195 °C and above. The reduction in viscosity, molecular weight as well as the decline of adsorption due to the removal of anionic side groups and the breakage of main chain structure were responsible for the deterioration of fluid loss capability.

From the analytic results in this paper, the following strategies was proposed and could be considered to enhance the thermal resistance of fluid loss agents in the future, (i) Increasing the relative molecular weight of the synthetic copolymer as much as possible to counteract the reduction caused by thermal degradation; (ii) innovating and synthesizing novel thermal-resistant monomers, such as incorporating some heat-resistant side groups (aromatic rings or rigid heterocyclic pyrrolidone structures) to protect anionic groups as much as possible; (iii) considering the introduction of antioxidants to delay the thermal degradation process of polymers.

## Data Availability

The original contributions presented in this study are included in the article. Further inquiries can be directed to the corresponding authors.
